# Experience of a Romanian Lyme Borreliosis Centre in the Multidisciplinary Management of Patients Evaluated for Suspected Lyme Neuroborreliosis

**DOI:** 10.3390/microorganisms14020264

**Published:** 2026-01-23

**Authors:** Violeta Briciu, Angela Monica Ionicǎ, Simona Mureşan, Astrid Binder, Cristina Cismaru, Oana Stan, Blanca Szolga, Cǎtǎlina Hǎpǎianu, Mirela Flonta, Mihaela Lupşe

**Affiliations:** 1 Department of Infectious Diseases and Epidemiology, “Iuliu Hațieganu” University of Medicine and Pharmacy, 23 Iuliu Moldovan, 400348 Cluj-Napoca, Romaniacristina.cismaru@umfcluj.ro (C.C.);; 2 Clinical Hospital of Infectious Diseases of Cluj-Napoca, 23 Iuliu Moldovan, 400348 Cluj-Napoca, Romania

**Keywords:** Lyme neuroborreliosis, differential diagnosis, multidisciplinary management

## Abstract

Lyme neuroborreliosis (LNB) may mimic other neurological diseases, while neurological diseases may be misdiagnosed as LNB. The aims of the study were to contribute to the knowledge regarding the epidemiology and clinical manifestations of LNB, discuss differential diagnosis, and compare characteristics in patients with and without LNB. We present patients evaluated for suspected LNB by the multidisciplinary team of a “Lyme Borreliosis Centre” in a highly endemic area in Romania. A retrospective study was performed between January 2011 and October 2023 on patients referred for suspected LNB based on neurological manifestations and positive serology for *Borrelia burgdorferi* antibodies using two-tier testing. A lumbar puncture was performed for diagnosis, and the European LNB definition was used for classification. Of three hundred and three LNB suspected patients, five (1.65%) were classified as definite LNB, eighty-three (27.39%) as possible LNB, and in two hundred and fifteen patients (70.95%), LNB was excluded. Comparing the definite/possible to excluded LNB patients, there was no significant difference in neurological symptoms/manifestations. The patients presented fifty-one neurological, twelve rheumatological, and seven psychiatric diagnoses, with significantly more meningitis/encephalitis/myelitis diagnoses in the definite/possible LNB group, and more demyelinating disease and discopathy in the LNB-excluded group. Considering the complex differential diagnoses, access to laboratory diagnostics and multidisciplinary management should be available in centres that evaluate suspected LNB patients. Comparing results with data from the national surveillance system, we conclude that LNB is underdiagnosed/underreported in Romania.

## 1. Introduction

Lyme borreliosis can mimic various conditions, particularly rheumatological, neurological, and cutaneous, which is why it is called the ‘great imitator,’ similar to another spirochete disease: syphilis. Lyme neuroborreliosis (LNB) may mimic other neurological diseases, and patients suffering from neurological disorders may be misdiagnosed as LNB. Early LNB, with symptoms that persist for less than 6 months, may present with lymphocytic meningitis, Garin–Bujadoux–Bannwarth polymeningoradiculoneuritis, cranial nerve neuritis (mainly involving the facial nerve), myelitis, or encephalitis. Late LNB, with symptoms that persist for more than 6 months, may present as mononeuropathy, radiculopathy, polyneuropathy or cerebral vasculitis, chronic progressive Lyme encephalitis, or encephalomyelitis with tetraspastic syndrome [1]. Late peripheral neuritis is usually associated with Acrodermatitis chronica atrophicans, which is caused by *Borrelia afzelii* and involves the sensory nerves in the affected skin areas (typically the distal extremities). It is usually not associated with inflammation of the cerebrospinal fluid and is likely the result of direct extension of the borreliosis infection from the skin to the underlying cutaneous nerves [2]. The serodiagnosis of Lyme borreliosis is based on a two-tier strategy (two-tier test): a screening test using an immunoenzymatic technique (ELISA), followed, if positive, by a confirmatory test with a Western blot technique [3]. According to the European guidelines, clinical suspicion of LNB due to neurological symptomatology in the absence of other causes should lead to cerebrospinal fluid (CSF) investigation for the presence of pleocytosis and simultaneous quantification of specific anti-*Borrelia (B.) burgdorferi* sensu lato antibodies and total antibodies in the blood and in the CSF to calculate the intrathecal antibodies index (IAI) [1,3,4]. Patients investigated for suspected LNB may present multiple subjective symptoms, such as asthenia, paraesthesia, headaches, irritability, depression, and memory disorders or inability to concentrate.

Even though LNB was included in 2018 in the list of diseases under EU epidemiological surveillance [5], diagnostic difficulties, under/overreporting, and different laboratory methods used remain important issues for LNB diagnosis and surveillance in Europe [6]. Public health surveillance for Lyme borreliosis (LB) in Romania began in 2007, with statutory reporting of LB cases by physicians to county health officers, who then report LB cases to the National Institute of Public Health [7]. The National Institute of Public Health publishes annual LB surveillance reports, including clinical manifestations of LB, with data available at the county level. However, the real number of LNB patients in Romania may be underreported by the passive surveillance system, and according to the most recently published data, there were three cases of possible LNB reported at the country level in 2023 [8].

Cluj County is an endemic area for LB [8], and a previous study on ticks collected from patients in 2010 showed an 11.1% prevalence rate of *B. burgdorferi* infection, with the presence of mainly *B. afzelii*, as well as of *B. garinii*, *B. burgdorferi* sensu stricto, *B. valaisiana*, and *B. lusitaniae* [9]. A countrywide study performed in 2010 and 2011 on 12,221 ticks, including sites from Cluj County, showed an overall prevalence of infection in questing ticks of 1.4%, but with a site from Cluj County showing a prevalence of more than 5% [10]. As the latest epidemiological study carried out in 2020 reported a seropositivity rate of 4% in blood donors from Cluj County (IgM or IgG antibodies by Western blot) [11], access to CSF investigations and differential diagnosis with other neurological diseases by a multidisciplinary team is important to rule out overdiagnosis of LNB, as serology or IAI may remain positive after treatment. Inappropriate attribution of LNB often leads to unnecessary prolonged antibiotic therapies that may be associated with adverse events in the absence of any benefit to the patient and increase the risk of global antimicrobial resistance [12].

The aim of the current study was to present data on epidemiology, clinical manifestations, and differential diagnosis in patients investigated for suspected LNB, and to compare characteristics between patients with definite or probable LNB and patients with excluded LNB. The manifestations in patients with definite LNB will be underlined. The study was performed in an academic infectious diseases hospital, where a “Lyme Borreliosis Centre” was founded in 2010, offering management for suspected LB patients by a multidisciplinary team (infectious disease specialist, microbiologist, neurologist, rheumatologist, ophthalmologist, psychiatrist, and cardiologist) similar to other European academic referral centres [13,14,15,16].

## 2. Materials and Methods

A retrospective study was performed in the Clinical Hospital for Infectious Diseases Cluj-Napoca, Romania, on all patients hospitalised for suspected LNB due to (1) neurological symptomatology and (2) a positive two-tier test (TTT) for anti-*B. burgdorferi* antibodies. A lumbar puncture was performed for diagnosis in all patients to detect pleocytosis and intrathecal anti-*B. burgdorferi* antibody production.

Patients referred for suspected LNB between 1 January 2011 and 31 October 2023 were evaluated by the infectious diseases specialists, who performed lumbar puncture, while neurological, rheumatological, and psychiatric consultations were performed when needed. A positive two-tier test consisted of a positive/borderline ELISA test and a positive/borderline Western blot test performed to detect IgM and IgG anti-*B. burgdorferi* antibodies, and results of these tests were collected from patients’ files, as these tests were performed before hospitalisation with the suspicion of LNB.

The IAI was calculated using (1) *Borrelia afzelii* + VlsE IgG and *Borrelia afzelii* IgM (Genzyme Virotech GmbH, Dietzenbach, Germany) in 2011; (2) Anti-*Borrelia* ELISA (IgM) and Anti-*Borrelia* plus VlsE ELISA (IgG) between 2012 and 2019; (3) recomWell *Borrelia* IgM and recomWell *Borrelia* IgG (Mikrogen Diagnostik, Germany) between 2020 and 2022; and (4) EIA *Borrelia* recombinant IgG and EIA *Borrelia* recombinant IgGM (TestLine Clinical Diagnostics, Brno, Czech Republic) in 2023. An IAI < 1.3 indicates no production of specific anti-*Borrelia* antibodies in the CSF, a value > 1.5 indicates a local production of specific antibodies and supports the diagnostic of LNB, while a value between 1.3 and 1.5 is considered borderline.

The case definition of LNB according to the European guideline [1], introduced into the Lyme Borreliosis Centre diagnosis protocol in 2010, was as follows: (1) neurological symptoms suggestive of LNB without other obvious causes; (2) CSF pleocytosis; and (3) intrathecal anti-*B. burgdorferi* antibody production (i.e., IAI > 1.5).

Definite LNB: All three criteria fulfilled.

Possible LNB: Two criteria fulfilled.

According to the guideline published by the Commission of the German Neurological Society, patients classified as possible LNB present a typical clinical picture (cranial nerve deficits, meningitis/meningoradiculitis, focal neurological deficits); *Borrelia*-specific IgG and/or IgM antibodies in serum; CSF findings not available/spinal tap not performed; and differentiation from other causes [4]. Based on this classification, which includes real-life situations where CSF findings may not be available, in our study, patients with detection of anti-*B. burgdorferi* antibodies in CSF and no further calculation of IAI (due to insufficient CSF or temporary laboratory incapacity for testing total IgM or IgG in CSF) were also classified as possible LNB, as were patients with borderline IAI (1.3–1.5).

Data were collected from the patients’ medical records at discharge. We obtained demographic, epidemiological, and clinical data; neurological, rheumatological, and psychiatric diagnoses (when consultation was performed at the recommendation of the infectious diseases specialist); IgM and IgG against *B. burgdorferi* by ELISA and Western blot tests; CSF parameters; results of cerebral MRI (when available); and discharge diagnosis. The final case classification (definite LNB, possible LNB, and excluded LNB) was performed by the principal study investigator in accordance with case definition. The neurological and rheumatological diagnoses were performed by specialists from the multidisciplinary team based on history and clinical and paraclinical examinations (biological and imaging when needed). As patients’ hospitalisation was mainly for diagnostic evaluation, no data on patients’ antibiotic therapy (ceftriaxone administered ambulatory when indicated) or clinical evolution were collected.

The statistical analyses were performed using EpiInfo 7 software (CDC, Atlanta, GA, USA). All qualitative variables were summarised as counts (n) and percentages (%), while quantitative variables were described using medians and interquartile ranges (IQR). The frequency and prevalence were calculated, and differences between groups were assessed by means of chi-square testing for categorical variables, the Mann–Whitney U test for continuous variables, and the level of significance was 0.05.

## 3. Results

A total of three hundred and three patients were included: one hundred and eighty-eight females (62.04%) and one hundred and fifteen males (37.95%), two hundred and thirty-one with urban residency (76.23%), with ages ranging between 4 and 81 years (median = 41 [IQR: 32–52]), including six children and two hundred and ninety-seven adults. The median duration of hospitalisation was 5 days (IQR: 2–14).

Serology results are presented in Table 1, and CSF analysis results are presented in Table 2. One patient was evaluated only for the presence of IgM in CSF, with a negative result, and is not included in the data in Table 2.

In total, 59 patients presented pleocytosis. Among them, twenty-six had anti-*B. burgdorferi* antibodies in the CSF: sixteen patients with only IgG antibodies, five with only IgM antibodies, and five with both IgM and IgG antibodies. The final patients’ classification, according to the previously mentioned criteria, was as follows: five patients with definite LNB (one child and four adults), eighty-three patients with possible LNB (one child and eighty-two adults), and in the remaining two hundred and fifteen patients, LNB was excluded (Supplementary Material).

**Figure 1:**
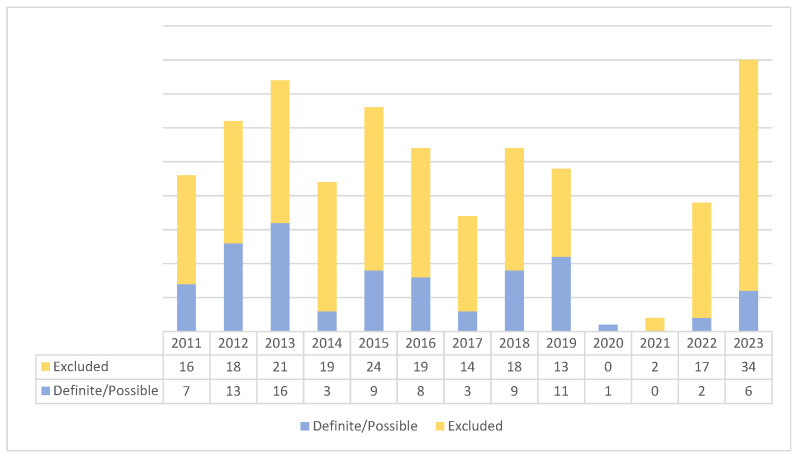
Patients’ classification and distribution by years.

The distribution of investigated patients during the study interval varied from a maximum of forty cases in 2023 to a minimum of one case during the 2020 COVID-19 pandemic year. Suspected and definite/possible patients’ distributions during the study interval are presented in Figure 1.

### 3.1. Comparison Between Patients with Definite/Possible LNB and Excluded LNB

Comparison of demographic and clinical characteristics between patients with definite/possible LNB and patients without LNB is presented in Table 3.

The statistical analysis showed a significantly higher prevalence of definite/possible LNB in males, as well as a significantly higher median duration of hospitalisation in the definite/possible LNB group.

### 3.2. Neurological Diagnosis

Overall, 211 patients (69.63%) presented at least one neurological diagnosis, of whom 55 had definite/possible LNB (Table 3). Patients were included in fifty-one neurological diagnoses/syndromes, with up to five diagnoses per patient, of which nineteen were present in both groups, twenty-three were associated only with patients with excluded LNB, and nine were associated only with patients with definite/possible LNB (Supplementary Material).

The distribution of the 19 neurological diagnoses present in both groups is presented in Table 4. The diagnosis of meningitis/encephalitis/myelitis was significantly associated with definite/possible LNB (*p* = 0.0002), while demyelinating disease was significantly associated with excluded LNB (*p* = 0.02).

### 3.3. Adult LNB

Four adult patients were classified as definite LNB (Table 5).

### 3.4. Paediatric LNB

Six children (under 18 years old) were evaluated for suspected LNB, and one definite and one possible LNB case were diagnosed.

The definite LNB paediatric patient was a 4-year-old boy, with rural residency and no known history of tick bite, who presented in the month of May with fever, somnolence alternating with psychomotor agitation, headache, vomiting, acute hemiparesis, expressive aphasia, eyelid myoclonia, and EEG abnormalities. Cerebral MRI showed diffuse oedema but no ischemic stroke in arterial territories, and EEG showed diffuse slowing with fronto-central spike waves. The neurological consultation revealed hemiparesis, psychomotor agitation, and frequent blinks, most likely epileptic seizures. As lumbar puncture was postponed due to cerebral oedema until the 13th day of ceftriaxone therapy, no pleocytosis was present; however, an IgM IAI of 16 was found, with no IgG in the CSF (while serum antibodies were IgM positive by both ELISA and Western blot tests). Due to the delayed lumbar puncture, he was classified as definite LNB even in the absence of pleocytosis, which was explained as a decrease in cell count due to the 13 days of antibiotic therapy. Clinical evolution was favourable under 28 days of ceftriaxone therapy, and although slight speech disorder and EEG features suggestive of absence seizures were still present at discharge, at the 3 month follow-up, no motor or speech disorders were present, and EEG was normal. The LNB-possible paediatric cases were diagnosed with acute meningoencephalitis, central right facial palsy, and cerebral ataxia.

### 3.5. Rheumatological Diagnosis

Seventy-seven patients (25.41% of the study group) presented a rheumatological diagnosis, including fifteen with definite/possible LNB and sixty-two with excluded LNB patients (Table 6), with up to three diagnoses per patient. The frequency of rheumatological conditions was significantly higher in patients with excluded LNB (statistically significant; *p* = 0.04). Overall, twelve rheumatological diagnoses were described, of which six were common to both groups, with the remainder described exclusively in excluded LNB patients (Supplementary Material).

### 3.6. Psychiatric Diagnosis

Sixty-five patients (21.45% of the study group) presented a psychiatric diagnosis, including sixteen with definite/possible LNB and forty-nine with excluded LNB patients (Table 7). Seven psychiatric diagnoses were described, of which four were common to both groups, with the remainder exclusively in excluded LNB patients (Supplementary Material).

Therapy for the neurological, rheumatological, or psychiatric conditions was recommended by the specialists and included in each patient’s individual management.

## 4. Discussion

This 12 year mono-centre retrospective observational study on 303 patients evaluated for suspected LNB based on neurological manifestations and a positive TTT for anti-*B. burgdorferi* antibodies underlines the diversity of differential neurological diagnoses, the association of rheumatological and psychiatric conditions, and the need for a multidisciplinary team for patient consultation. More females than males were evaluated for suspected LNB, and more patients were from urban areas. The study identified definite/possible LNB in 29.05% of patients (n = 88), while in the majority (n = 215; 70.95%) LNB was excluded.

The demographic data show a higher prevalence of definite/possible LNB in males. Few studies have analysed sex-related differences [17], and although the prevalence of early Lyme disease appears to be relatively equal between sexes, late LB with objective neurologic or rheumatologic findings appears to be more common in males than in females, while subjective syndromes such as post-treatment Lyme disease syndrome (PTLDS) appear to be more commonly reported in females than in males, with possible explanations in the difference in immune response between males and females following infection [18].

No difference in residence between the two groups of patients (definite/possible LNB and excluded LNB) was found, which is similar to a recent study that evaluated the seroprevalence of anti-*B. burgdorferi* antibodies in blood donors from the same region [11]. A higher age, with a median of 43 years, was found in the definite/possible LNB group compared to the excluded LNB group, but it was younger than in other retrospective studies published on LNB patients [19,20].

A notable percentage of patients from the definite/possible LNB group did not record a tick bite (7.95%), underlining the importance of testing for *B. burgdorferi* infection in suspected patients from endemic areas even in the absence of a known tick bite. On the other hand, a higher percentage of patients from the definite/possible LNB group recorded a tick bite compared to the excluded LNB group, although it was not statistically significant.

Patients were referred with the suspicion of LNB for further CSF investigation based on clinical decision (epidemiology, symptomatology, exclusion of other diseases, a TTT for *B. burgdorferi* antibodies), as it is known that the diagnostic specificity of serum antibody tests is low because seropositivity in the normal population ranges from 5% to >20% [1]. As presented in Table 3, IgM antibodies both by ELISA and Western blot were significantly more prevalent in the excluded LNB group, while IgG antibodies both by ELISA and Western blot were significantly more prevalent in the definite/possible LNB group. The high frequency of IgM antibodies in the excluded LNB group supports the data on the high frequency of false-positive IgM antibodies even on immunoblots in clinical practice [21].

Regarding the type of *B. burgdorferi* antibodies produced in the CSF, we found both IgM and IgG antibodies (six patients had positive IgM IAI, while two patients had both IgM and IgG IAI). Krogen et al. [22], in a retrospective Danish cohort of 544 patients with a positive *B. burgdorferi* IAI (IgM IAI, IgG IAI, or both), showed that Intrathecal Borrelia-specific antibody production did not follow the typical immune response of initial IgM production followed by IgG production, and diagnosis of LNB stage should not be based on the type of antibodies found in the cerebrospinal fluid.

Our study underlines the diverse neurological symptomatology described both in the definite/possible LNB and the excluded LNB group, supporting the role of the multidisciplinary team for patient management. No statistically significant difference in symptoms like headache, paraesthesia, neuropathic pain, memory and speech disorders, facial palsy or other cranial nerve involvement, vertigo, gait disorder, altered mental state, motor impairment, or fatigue between the two groups was found, underlining the non-specific and highly diverse clinical manifestations in LNB. Frahier et al. [20], in a study comparing 45 LNB patients to 100 excluded LNB patients, found no difference in neurological symptomatology except for facial palsy, radiculopathy, and neuropathic pain. Regarding symptom duration, it was significantly higher (>6 months) in the LNB-excluded group, suggesting that patients with definite LNB are presenting earlier for investigations.

The neurological diagnosis in patients evaluated for suspected LNB is very diverse, and we have identified, overall, 51 neurological diagnoses/syndromes, with 19 diagnoses/syndromes common to both groups and 23 diagnoses/syndromes only in the excluded LNB group. The diagnosis of meningitis/encephalitis/myelitis was significantly associated with definite/possible LNB. The French guideline for LB presents possible causes of persistent symptoms after documented or suspected Lyme borreliosis, and among the neurological diseases are found Parkinson’s disease, multiple sclerosis, amyotrophic lateral sclerosis, epilepsy, myasthenia, migraine, dementia, or post-traumatic encephalopathy [3].

Multiple sclerosis (MS) was diagnosed in both groups (five cases in definite/possible LNB and fifteen in excluded LNB). One of the patients diagnosed as definite LNB was under observation for suspected MS based on cerebral and spinal MRI results (and no improvement after antibiotic therapy), and four patients from the possible LNB group were also diagnosed with MS. Ranjeevan et al. [23] also reported a patient with definite LNB who had brain MRI and cerebrospinal fluid findings compatible with MS, underlining that a diagnosis of LNB does not rule out a diagnosis of multiple sclerosis. Based on the observation that both LB and MS are associated with an abnormal immune response, and that some people with MS have reported a prior history of LB or exposure to ticks, there was the theory that *B. burgdorferi* infection may trigger an autoimmune response in some people, leading to the development of MS, but studies have reported conflicting results [24].

A retrospective observational cohort study that evaluated patients referred for LB to a Mid-Atlantic academic centre from 2000 to 2013 identified that, in patients who lacked evidence for *B. burgdorferi* infection, the leading diagnoses were anxiety/depression, fibromyalgia, chronic fatigue syndrome, migraine disorder, osteoarthritis, and sleep disorder/apnea, while less frequent diseases included multiple sclerosis, malignancy, Parkinson’s disease, sarcoidosis, or amyotrophic lateral sclerosis [25]. In the present study, amyotrophic lateral sclerosis (ALS) was diagnosed in five patients with excluded LNB, and a large case–control study on 491 patients with ALS and 982 controls provides evidence for a lack of association between anti-*B. burgdorferi* antibodies and ALS [26]. Rare diseases such as Clippers syndrome, Parsonage Turner syndrome, or Melkersson–Rosenthal syndrome were also diagnosed in the study group, further underlining the need for a multidisciplinary approach for differential diagnosis in rare diseases. Pfefferkorn et al. [27] reported five cases of brainstem encephalitis in LNB, arguing for a typical clinical course with distinct MRI findings in a subset of affected patients, with the “Tarsier Sign” in coronal FLAIR imaging as the most prominent feature. The patient suspected of Clippers syndrome in our cohort was classified as definite LNB; MRI showed multiple focal lesions with a tendency to confluence at the pontine level, without contrast enhancement. Clippers syndrome (chronic lymphocytic inflammation with pontine perivascular enhancement responsive to steroids) is an entity thought to represent immune-mediated inflammation. Lindland et al. [28] reported a similar LNB case with MRI modifications suggestive of Clippers syndrome that responded to antibiotic therapy.

Parkinson-like symptoms have been associated with *B. burgdorferi* infection. Hudasch et al. [29] reported from Germany a case of LNB with movement disorders mimicking Parkinson’s disease that improved after antibiotic therapy, while Cassarino et al. [30] reported a case that declined despite treatment, with progressive disability. In the study group, Parkinson’s disease was diagnosed in two excluded LNB patients, with no cases in the definite/possible LNB group.

Two patients from the group of definite/possible LNB had an associated diagnosis of cerebral vasculitis. Acute stroke-like symptoms caused by cerebral vasculitis are rare and have been documented in case reports [31,32]. Five patients from the possible LNB group presented with a stroke that preceded the hospitalisation for suspected LNB.

Demyelinating lesions were found both in the LNB-definite/possible and LNB-excluded groups, with a significant difference for patients with excluded LNB, supporting previously published data that MRI findings of the brain are rare in LNB, and white matter lesions may be incidental in LNB patients [33]. The role of MRI in LNB consists of ruling out other causes of neurological symptoms. Volk et al. [34] showed in a retrospective study on 35 LNB patients that MRI findings were heterogeneous, showing longitudinally extensive myelitis, peri-medullar leptomeningeal enhancement, ponto-mesencephalic lesions, or cerebral vasculitis, and no clear pattern of MRI findings in LNB could be identified. No systematic evaluation by MRI was performed in all patients from the present study.

There was only a small number of paediatric patients investigated for suspected LNB (n = 6, 1.98%), with two definite/possible paediatric LNB, while a retrospective study of LNB over a 14 year interval in Sweden described a more than double incidence of LNB in children compared with adults (111 paediatric LNB) [35]. In France, between 2001 and 2012, nine paediatric LNB cases were collected by the French nationwide active surveillance network of paediatric bacterial meningitis, representing 0.2% of all meningitis cases collected by the network [36]. The patient classified as definite LNB in this study presented with encephalitis, hemiparesis, expressive aphasia and epileptic seizures. LNB presenting with encephalitis is scarcely described, and even more rarely described in children. A review of all articles published until 2021 on definite LNB and definite/possible encephalitis identified 45 patients from 18 countries spanning 35 years, out of which eight were children [37]. The exact mechanisms by which *B. burgdorferi* may cause acute focal neurologic deficits in the absence of vasculitic stroke, like in our patient, are still not understood [29]. LNB should be considered in children with clinical manifestations of acute encephalitis and epilepsy, even in the absence of a tick bite history.

Previous studies have reported a low rate of LB confirmation (up to 15%) in patients referred for suspected LB and managed through a multidisciplinary approach [14,15,16,25]. Regarding LNB confirmation in patients consulted for suspected LNB, a recently published French study on 155 patients identified definite/probable LNB in 45 patients (29%), similar to the present study, while confirmation of another neurological disease was established in 29% of excluded LNB patients [20]. In patients with neurological symptoms referred for suspicion of LNB, especially since serum-specific antibodies might be present (as a marker of a previous infection [38] or false-positive antibodies [21]), lumbar puncture results make it easier for patients and their families to accept the absence of antibiotic prescriptions.

The current study underlines the diversity of rheumatological diagnoses established both in the definite/possible LNB and excluded LNB groups, with no differences between common diagnoses except for discopathy, which was significantly more frequent in the excluded LNB group. Most diagnoses were represented by degenerative rheumatic disorders like arthrosis, spondylosis, or discopathy, while inflammatory diseases like arthritis, dermatomyositis, or polymyositis were diagnosed only in the excluded LNB group. These pathologies were not associated with *B. burgdorferi* infection, but it is important that they be diagnosed and managed with specific treatment, different from antibiotic therapy, as recommended by patient-centred care protocols [16]. Frahier et al. [20] established a rheumatological diagnosis in 19% of the LNB-excluded group, while in the present study, rheumatological diagnoses were established for 25.41% of the overall study group and 28.84% of the excluded LNB group, with the data underlining the need for a rheumatological consultation in suspected LNB patients.

There is no strong evidence supporting Lyme borreliosis’ role in long-term cognitive or psychiatric disorders, with some studies suggesting an increased risk of depression, anxiety, sleep disturbances, and other mental health conditions, while others find no such association [39]. No significant differences between the definite/possible LNB and excluded LNB groups were found in this study regarding overall psychiatric disorders, depressive disorder, anxiety disorder, anxiety-depressive disorder, or somatization disorder, and this underlines the need for a psychiatric consultation in suspected LNB patients, as 21.45% of the patients had an associated psychiatric diagnosis.

Apart from overdiagnosis in clinical practice [25], under-reporting is a recognised issue for LB surveillance in Europe, with heterogeneous surveillance across different countries; countries like Austria have no public health surveillance for LB, whereas others, such as Lithuania and Romania, have national surveillance [40]. Our data show that more cases were diagnosed with possible LNB in our centre in 2023/2019/2018/2017 (six patients, eleven patients, nine patients, three patients) than the total number reported at the country level in the same years (three cases, two cases, five cases, one case) [8], underlining the under-reporting of LNB by the passive surveillance system in Romania. Angulo et al. [41] showed there is a higher incidence of symptomatic *B. burgdorferi* infection than is reported through public health surveillance for LB in Romania, with an under-detection multiplier of 10.5. Regarding published data from Romania on LNB, we found one case series study on LNB including 50 patients over three years from central Romania [42] and a previously published study from our centre on 42 patients investigated for suspected LNB [43].

The main study limitation is the absence of IAI calculation in a large number of patients with detectable *B. burgdorferi* antibodies in CSF, further classified as possible LNB. Another limitation of the retrospective study is the absence of patients’ neurological follow-up, as this was mainly performed in the neurology department after discharge from the infectious diseases department. On the other hand, the complex neurological diagnoses/syndromes at baseline underline the real-life diagnosis difficulties in patients hospitalised for suspected LNB. Being a retrospective study on discharge files of patients evaluated for suspected LNB, we also do not present a prospective clinical evolution of the definite/possible LNB patients under ambulatory antibiotic therapy. The strengths are represented by the large number of patients investigated, being the largest study from Romania that provides epidemiological and clinical data on LNB using the European guidelines’ definition and a multidisciplinary approach.

## 5. Conclusions

This is the largest study from Romania that presents patients investigated for suspected LNB in a Lyme borreliosis Centre, managed using a holistic approach. During a 12 year interval, out of 303 suspected LNB patients, 29.05% were classified as definite/possible LNB. No significant association between definite/possible LNB and neurological symptoms/manifestation, or a specific neurological diagnosis, was found. Considering the complex clinical picture and the variety of differential diagnosis, a multidisciplinary management and access to CSF laboratory tests for LNB should be considered in all centres that evaluate patients for suspected LNB. Our data suggest that in Romania LNB might be under-diagnosed and under-reported to the national passive surveillance system, while overdiagnosis might occur in the absence of access to laboratory diagnosis for CSF investigation and in the absence of multidisciplinary management for differential diagnoses.

## Figures and Tables

**Table 1:** Results of serum anti-*Borrelia* antibodies testing by ELISA and Western blot tests.

		IgG								
		E− W−	E− W±	E− W+	E± W−	E± W±	E± W+	E+ W−	E+ W±	E+ W+
IgM	E− W−	−	−	−	−	1	2	−	2	43
	E− W±	−	−	−	−	−	−	−	1	−
	E− W+	−	−	−	−	1	−	2	2	1
	E± W−	−	−	2	−	−	−	−	1	2
	E± W±	2	−	−	−	−	−	−	−	1
	E± W+	9	1	2	−	−	−	−	−	2
	E+ W−	−	7	17	−	1	1	−	5	23
	E+ W±	3	1	5	−	−	1	−	−	4
	E+ W+	100	4	7	4	−	2	11	5	25

E: ELISA; W: Western blot; +: positive; ±: borderline (grey zone); −: negative.

**Table 2:** Results of anti-*Borrelia* antibody presence in patient CSF samples and IAI.

		IgG				
		Absent	Present IAI < 1.3	Present 1.3 < IAI < 1.5	Present IAI > 1.5	Present IAI not Calculated
IgM	Absent	193	21	3	10	52
	Present IAI < 1.3	-	1	-	-	-
	Present IAI > 1.5	4	-	-	2	-
	Present IAI not calculated	10	-	-	-	6

**Table 3:** Comparison of characteristics between patients with definite/possible LNB and excluded LNB. Statistically significant *p*-values are highlighted in bold.

Variable	Subcategories	Definite/Possible LNB (n = 88; 29.05%)	Excluded LNB (n = 215; 70.95%)	*p*-Value
Sex	M	44; 50%	71; 33.02%	**0.006**
	F	44; 50%	144; 66.98%	
Residence	Rural	27; 30.68%	45; 20.93%	0.075
	Urban	61; 60.32%	170; 79.07%	
Median age	-	43	41	0.274
Median hospitalisation duration (days)	-	8	4	**0.001**
History of tick bite	no	7; 7.95%	29; 13.62	0.2
	yes	35; 39.77%	75; 35.21%	
	no data	46; 52.27%	111; 51.63%	-
Symptoms/neurological manifestations	headache	38; 43.18%	90; 41.86%	0.89
	paraesthesia	38; 43.18%	98; 45.58%	0.79
	neuropathic pain	7; 7.95%	9; 4.19%	0.25
	memory disorder	9; 10.23%	19; 8.84%	0.66
	speech disorder	7; 7.95%	16; 7.44%	0.8
	facial palsy	6; 6.82%	7; 3.26%	0.11
	other cranial nerve	7; 7.95%	23; 10.7%	0.66
	vertigo	26; 29.55%	77; 35.81%	0.34
	gait disorder/vertigo	12; 13.64%	29; 13.49%	0.85
	altered mental state	8; 9.09%	18; 8.37%	0.82
	motor impairment	15; 17.05%	41; 19.07%	0.87
	fatigue	28; 31.82%	54; 25.12%	0.25
Symptoms duration	<6 months	43; 48.86%	66; 30.7%	**0.009**
	>6 months	35; 39.77%	112; 52.09%	
	no data	10; 11.36%	37; 17.21%	-
Serology	IgM (ELISA) positive or borderline	65; 73.86%	183; 85.12%	**0.03**
	IgG (ELISA) positive or borderline	64; 72.73%	79; 36.92%	**<0.0001**
	IgM (WB) positive or borderline	42; 47.73%	154; 71.96%	**0.0001**
	IgG (WB) positive or borderline	72; 81.82%	100; 46.51%	**<0.0001**

**Table 4:** Neurological diagnoses/syndromes in definite/possible LNB and excluded LNB patients. Statistically significant *p*-values are highlighted in bold.

Diagnosis/Syndrome	Definite/Possible LNB		Excluded LNB		*p*-Value
	n	%	n	%	
overall	55	62.5	156	72.56	0.09
Meningitis/encephalitis/myelitis	16	18.18	9	4.19	**0.0002**
demyelinating disease	7	7.95	40	18.6	**0.02**
peripheral neuropathy	7	7.95	16	7.44	1
tetra/hemi/paraparesis	5	5.68	19	8.84	0.48
multiple sclerosis (MS)	5	5.68	15	6.98	0.8
cranial nerve paresis	5	5.68	10	4.65	0.77
stroke	5	5.68	7	3.26	0.33
seizures	4	4.55	9	4.19	1
cephalalgia syndrome	3	3.41	18	8.37	0.14
vertigo	3	3.41	18	8.37	0.14
vestibular syndrome	3	3.41	7	3.26	1
hypoacusis	2	2.27	12	5.58	0.36
cerebral microangiopathy	2	2.27	5	2.33	1
cerebral vasculitis	2	2.27	4	1.86	1
paraesthesia	1	1.14	7	3.26	0.44
cerebellar syndrome	1	1.14	3	1.4	1
neuralgia	1	1.14	3	1.4	1
neuritis	1	1.14	2	0.93	1
syncope	1	1.14	1	0.47	0.49

**Table 5:** Description of the four adult patients with definite LNB.

Patient	Description
1.	A 52-year-old male patient presented with daily occipital headache with a 6 months’ duration, later associated parietal and occipital paraesthesia, with cervical irradiation. The patient recorded a tick bite two years prior. Brain MRI showed cerebral microangiopathy, periventricular leukoaraiosis, incipient cortico-subcortical, and cerebellar–cerebral atrophy. The neurological consultation established the diagnosis of cerebral microangiopathy, the rheumatological consultation established the diagnosis of cervico-occipital neuralgia and the psychiatric consultation established the diagnosis of adjustment disorder with anxiety. CSF investigation showed 21 cells/mmc, and the *B. burgdorferi* IgG IAI was 2.18. The patient was transferred in a local hospital near his residency for initiation of ceftriaxone therapy at 2 g/day for 21 days.
2.	A 55-year-old female patient presented 3 months after a tick bite and 2 months after recording an erythema migrans-like lesion (not evaluated in a medical department), with intense anterior and posterior chest pain and right peripheral facial palsy. Cerebral MRI showed right frontal demyelinating lesions in the deep white matter and left frontal subcortical region measuring up to 5–7 mm with no contrast intake; findings were non-specific (more likely microangiopathic). The neurological consultation established the diagnosis of facial palsy and cerebral microangiopathy. CSF investigation showed 175 cells/mmc; the *B. burgdorferi* IgM IAI was 4.39, and the IgG IAI was 40.78. The patient followed therapy with ceftriaxone at 2 g/day for 28 days with complete resolution of the thoracic pain, partial resolution of the facial palsy, and recommendation to continue facial kinetotherapy for rehabilitation and neurological follow-up.
3.	A 50-year-old female patient presented with a 9 year history of dizziness, gait disorders, distal paraesthesia in the limbs, spastic tetraparesis with predominance of paraparesis, urinary incontinence, and emotional lability. The patient was frequently evaluated in the neurological department. Brain and cervical MRI detected demyelinating lesions of the brain and spine. The neurological consultation suspected the diagnosis of multiple sclerosis, but referred the patient for suspected LNB due to the presence of anti-*B. burgdorferi* IgM serum antibodies (ELISA and Western blot tests). CSF investigation showed 12 cells/mmc, and the *B. burgdorferi* IgG IAI was 4.67. The patient was recommended therapy with ceftriaxone at 2 g/day for 21 days. She was reevaluated in the infectious diseases department after 6 months with persistence of the symptomatology. Repeated lumbar puncture showed no presence of *B. burgdorferi* antibodies in the CSF, and she was referred for neurological management with the suspicion of multiple sclerosis.
4.	A 54-year-old female patient presented with symptoms that started insidiously 9 months prior, including headaches, cervical muscle pain, severe fatigue, and later diplopia. A previous hospitalization in the neurological department excluded neurosarcoidosis and bacterial and viral meningitis (multiplex PCR panel negative; PCR for Mycobacterium tuberculosis negative) but confirmed pleocytosis by lumbar puncture (568 cells/mm3, mainly lymphocytes). Cerebral MRI showed multiple focal lesions with a tendency to confluence at the pontine level, with no contrast enhancement; a focal lesion in the tectal plate, possibly a glioma; and non-specific demyelinating lesions. Clippers syndrome or LNB was suspected, and the patient was referred for further investigations. CSF investigation showed 12 cells/mmc; the *B. burgdorferi* IgM IAI was 2.14, and the IgG IAI was 10.35. The patient was recommended therapy with ceftriaxone at 2 g/day for 28 days with remission of neurological complaints at 6 months’ follow-up.

**Table 6:** Rheumatological diagnosis in definite/possible LNB and excluded LNB patients. Statistically significant *p*-values are highlighted in bold.

Diagnostic	Definite/Possible LNB		Excluded LNB		*p* -Value
	n	%	n	%	
overall	15	17.05	62	28.84	**0.04**
arthrosis	7	7.95	14	6.51	0.62
spondilosis	4	4.55	13	6.05	0.78
dyscopathy	3	3.41	27	12.56	**0.02**
fibromyalgia	1	1.14	5	2.33	0.67
antiphospholypidic syndrome	1	1.14	1	0.47	0.49
epicondylitis	1	1.14	1	0.47	0.49

**Table 7:** Psychiatric diagnosis in definite/possible LNB and excluded LNB patients.

Diagnostic	Definite/Possible LNB		Excluded LNB		*p* -Value
	n	%	n	%	
overall	16	18.18	49	22.79	0.44
depressive disorder	7	7.95	15	6.98	0.8
anxiety disorder	4	4.55	16	7.44	0.45
anxiety-depressive disorder	4	4.55	12	5.58	1
somatization disorder	1	1.14	1	0.47	0.49

## Data Availability

The original contributions presented in the study are included in the article/Supplementary Material, further inquiries can be directed to the corresponding author.

## Supplementary Materials

The following supporting information can be downloaded at: https://www.mdpi.com/article/10.3390/microorganisms14020264/s1, Supplementary File S1: anonymized patient database.

## Abbreviations

The following abbreviations are used in this manuscript:

LNB	Lyme neuroborreliosis
LB	Lyme borreliosis
*B.*	*Borrelia*
ELISA	Enzyme-linked immunosorbent assay
WB	Western blot
CSF	Cerebrospinal fluid
IAI	Intrathecal antibodies index
TTT	Two-tier test
